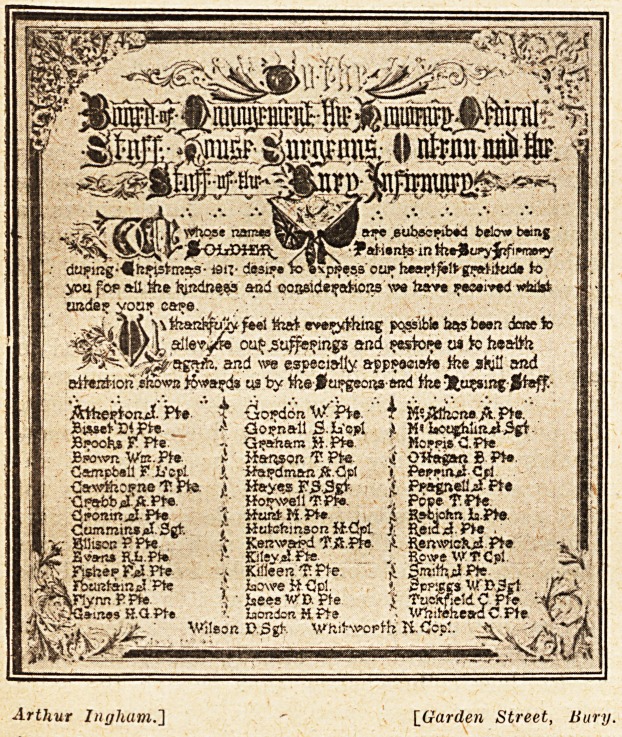# Hospital and Institutional News

**Published:** 1918-04-27

**Authors:** 


### HOSPITAL AND INSTITUTIONAL NEWS.

### IMPORTANT DEVELOPMENTS AT LEICESTER.

An important conference with Sir John Collie, Director of Medical Service of the Ministry of Pensions, and the Chairman and members of the board of the Leicester Royal Infirmary has ended in the definite decision to proceed at once with the establishment of an orthopædic department for the treatment of discharged disabled sailors and soldiers at the infirmary. The board has consented to erect in the grounds of the institution an in-patients' department to accommodate fifty patients. The work will be immediately proceeded with as soon as the Ministry gives the necessary building permit under the Defence of the Realm Act. Further, the Mayor of Leicester (Alderman Jonathan North, J.P., who was present at the conference) and the committee associated with him in the administration of the Wounded Heroes Fund have agreed to erect an out-patients' orthopædic department in connection with the infirmary. A further development of workshop accommodation is also projected. The Hospital has paid unceasing tribute to the patriotic service rendered to the country during the war by the voluntary hospitals in co-operating with the various Government departments.

### A NEW FINANCIAL POLICY.

It is not often either that a well-thought-out financial policy has so quickly proved itself as at Leicester. In the report of the annual meeting of the infirmary, which appeared in The Hospital of the 6th inst., mention was made of Mr. Alfred Corah's successful effort as Treasurer of the institution in securing a more adequate subscription list. Mr. Corah's idea was that if it were possible to set aside from annual subscriptions a definite sum each year to a reserve fund, the recourse to intermittent appeals for extensions would largely be avoided. In accordance with this view, the treasurer had increased the subscription list in the past two years by upwards of £3,000 per annum, and as there is little doubt that a similar result will be achieved this year the institution will be in the happy position of possessing a liquid cash reserve of £9,000, a sum which should be sufficient to provide for the important developments now decided upon. In view however, of the uncertainty in the cost of new buildings, it is by no means certain that the sum in question will wholly provide for the new departments, but the patriotic decision of the board to erect out of the accumulated funds what is really a war development will stimulate the local spirit of generosity in order to open the new department free of debt.

### TWO FRESH ENTERPRISES.

At the same annual meeting the decision was also announced to rebuild, whenever it is possible to do so, the only reconstructed wing of the institution as a memorial to the great services of Sir Edward Wood, the late chairman. Hence there arises a great opportunity for the residents of Leicestershire to set a double seal upon the work of Sir Edward Wood. Another local enterprise which greatly interested Sir John Collie on his visit to Leicester was the Home for Neurasthenic and Shell-shocked Soldiers, which, largely at his suggestion, the Mayor of Leicester established out of his Wounded Heroes Fund. This home is now in working order, and already progress has been made in the treatment of patients under the medical supervision of Colonel Astley Clarke. We commend the Mayor for the invaluable service which he is making of the fund raised under his auspices.

### THE COST OF PENSIONS.

Though one hears complaints of individual instances of official parsimony, the year's review of the work done by the Ministry of Pensions, just issued by the Select Committee of National Expenditure, should dispel any idea that such cases are of common occurrence. During the past financial year £23,000,000 has been expended, whilst the estimated expenditure for 1918-1919 is £41,500,000. The financial charge for pensions will be at its highest in the year following the termination of the war, after which it will show a gradual diminution. At present no fewer than 635,000 persons are receiving aid from the Department in some form, whilst 18,500 men are under special treatment and training. Three classes of case are causing much difficulty to the Department: men who were in ill-health at the time of enlistment and whose health has been further impaired by their period of military service, those similarly enlisted but in whose case there exists considerable doubt as to whether their service is actually responsible for their ill-health, and, lastly, those who in the future may fall into ill-health as a result of the hardships they have undergone. Confidence, however, may be felt that under the able direction of Mr. Hodge, the Minister of Pensions, no case will be allowed for long to go without a recognition of services rendered, wherever such a recognition is due.

### A POOR-LAW CRITICISM ON THE REFORM OF INSTITUTIONAL MEDICAL RELIEF.

Last week's issue of the *Poor Law Officers' Journal* contains a criticism of the article in our columns on "London's Institutional Medical Relief: Proposals for its Reform." Much of the criticism is devoted to the verbal quibbles a critic is driven to when he finds it difficult to bring forward arguments against views he dislikes. One sentence is worthy of quotation as showing the attitude of a leading apologist for our Poor-Law system to suggestions for its reform. Describing the scheme detailed in our columns as a "Hospital Utopia," he sums up: "All this is well enough, indeed, as a medical and surgical programme of the next to unattainable in a sort of Elysium for the sick and diseased. But how Dr. Thackray Parsons or any other medical man or officer can bring himself to the belief that it will be compassed by the transference of Poor-Law administration to the County Council will require more explanation than can be given by any person of experience in local administration." That is just the attitude experience has taught us to expect from most Poor-Law officials and Guardians. It is just because every great advance appears to them an unattainable Utopia, and that they cannot see the use of striving for it, that they have always lagged behind other agencies in their provision for the medical needs of the poor. If our critic will read our article carefully he will learn how the County Council administering relief over a large area, if actuated by a different spirit, will find no insuperable difficulty in reaching a goal the Poor-Law official mind finds unattainable.

### THE DANGER OF UNQUALIFIED PERSONS ON HOSPITAL STAFFS.

The difficulty in which hospitals are placed by the appointment of unqualified persons has been shown in the unfortunate occurrence of the death of an infant, aged eleven months, on the day after its discharge from the Royal South Hants and Southampton Hospital. The foster-mother and another woman declared that the child showed no improvement on its discharge, and at the adjourned inquest Dr. Omar, senior surgeon to the hospital, stated that he was inclined to attribute the death to natural causes. Dr. Omar, who has left Southampton because the place did not suit him, stated that the house physician saw the child when the witness did not, and that the house physician was unqualified. He added that there was no other qualified person, for the qualified woman doctor did not work in the wards. The two honorary physicians visited the hospital but rarely, and Mr. Bambridge, the unqualified house physician, was responsible for discharging the child. The Coroner remarked that the child had evidently been in the care of an unqualified person, and such a state of affairs should not pass without notice. The jury, in returning a verdict in accordance with the medical evidence, declared that the person on whose authority the child was discharged, when it was unfit to be discharged, deserved censure. We do not wish to be too hard on hospitals in days when there is a scarcity of qualified men, but at the same time it is essential to know what steps the Royal South Hants Hospital is taking to remedy a state of affairs which leaves the patients apparently without the regular care of any qualified person. Dr. Omar's frank admissions are very damaging, and the conditions thus revealed must be stopped.

### REPRESENTATION AND EFFICIENCY.

The question of the representation of the Worcester Hospital Saturday Committee upon the board of the General Infirmary has created some heart-searchings in the city. It has been found in the Midlands and the North, where great industrial centres abound, that if a representative is given for every lump sum subscribed the committee is over-weighted. And as representation is a much-prized right, the difficulty of avoiding inflated committees has been serious. It therefore seems to us that the proper course to pursue is to fix first of all the limits of a manageable executive committee, and then to give a fair proportion of representatives to any section of the public. There is no virtue in numbers; in fact, the larger a committee the worse, and the more undemocratic it usually is. The House of Commons is a flagrant example. If the measurable size of the executive committee is the first consideration, it should not be difficult to apportion representatives upon it, and where this is done the evil of constantly revising the amount of a subscription which secures a representative is avoided.

### THE HARDSHIPS OF THE TICKET SYSTEM.

Every instance of the decay of the ticket system is worth recording, for it is one which has no virtues to set against the drawbacks and, indeed, the cruelties which it entails. At the South Devon and East Cornwall Hospital Mr. J. H. Beckly has proposed its abolition and urged that the Plymouth hospitals should co-operate to end the nuisance as London, Manchester, and other big cities had done. He remarked that the most needy were often those who found tickets most difficult to secure, and often trudged round the town in vain in a humiliating and unsuccessful search. He asked why a central office could not be established to which applications could be made. His suggestion was supported by Mr. John Adams, and Sir Henry Lopes suggested that a conference should be held upon it. He also suggested various ways in which the hardships of the ticket system might be reduced; but the proper course to take is to abolish it altogether. The argument that the system is a source of income is negligible. Voluntary hospital finance has a far surer basis than the sale of petty patronage. The system only survives because people have grown accustomed to it; but the truth is that a ticket disgraces him that gives and him that has to tramp to find it.

### A GOVERNMENT CONCESSION.

In regard to the Government's attitude towards the treatment and training of disabled soldiers, an important concession appears to have been made. At the recent annual meeting of the Newcastle Royal Infirmary it was announced that the Government had promised to contribute the sum of £10,000 to the orthopædic centre at Newcastle.

### A COLLIERY OWNER'S SUGGESTION.

It is pleasant to see big employers setting an example to their workmen in regard to their hospital subscriptions. This appears to have occurred at Mansfield, where Mr. J. P. Houfton has offered, on behalf of the Bolsover Colliery Company, to increase its annual subscription by 50 per cent. if the workmen will raise their contribution by a similar proportion. Mr. Houfton has suggested that all the colliery owners of the district should follow suit. We hope that they will. If, however, their example is to bear full fruit, the annual subscription from each company should not be merely a round sum; it should be, like the workmen's, based on a definite calculation. The fair parallel to the workmen's penny a week should be a sum reckoned at so much per head for each person employed by each company. Were this plan adopted generally, as it has been in certain areas, the deficit on Mansfield and District Hospital would be a thing of the past.

### A PATIENTS' TESTIMONIAL.

A PLEASING ceremony took place at the Bury Infirmary recently, when the wounded soldier patients who were inmates at Christmas were so grateful for what was done for them, that they expressed their gratitude in the form of an illuminated address which now hangs on the walls of the board-room of the infirmary. The president of the institution received the testimonial on behalf of the board of management, and expressed appreciation for this token of gratitude. The testimonial

Board of Management, the Honorary Medical Staff, House Surgeons, Matron and the Staff of the Bury Infirmary.

We whose names are subscribed below being Soldier Patients in the Bury Infirmary during Christmas 1917 desire to express our heartfelt gratitude to you for all the kindness and considerations we have received whilst under your care.

We thankfully feel that everything possible has been done to alleviate our sufferings and restore us to health again, and we especially appreciate the skill and attention shown towards us by the Surgeons and the Nursing Staff.

| | | |
|---|---|---|
| Atherton J. Pte. | Gordon W. Pte. | McAlhone A. Pte. |
| Bisset Dd. Pte. | Gornall S. L'cpl | McLoughlin J. Sgt. |
| Brooks F. Pte. | Graham H. Pte. | Morris C. Pte. |
| Brown Wm. Pte. | Hanson T. Pte. | O'Hagan B. Pte. |
| Campbell F. L'cpl. | Hardman A. Cpl | Perrin J. Cpl |
| Cawthorne T. Pte. | Hayes F.S. Sgt. | Pragnell J. Pte |
| Crabb J. A. Pte. | Horwell T. Pte. | Pope T. Pte. |
| Cronin J. Pte. | Hunt M. Pte. | Robjohn L. Pte. |
| Cummins J. Sgt. | Hutchinson H. Cpl. | Reid J. Pte. |
| Ellison P. Pte. | Kenward T. A. Pte. | Renwick J. Pte |
| Evans R.L. Pte. | Kiley J. Pte. | Rowe W. T. Cpl. |
| Fisher F.J. Pte. | Killeen T. Pte. | Smith J. Pte. |
| Fountain J. Pte | Lowe H. Cpl. | Spriggs W. D. Sgt. |
| Flynn P. Pte. | Lees W. D. Pte. | Tuckfield C. Pte. |
| Gaines H. G. Pte. | London H. Pte. | Whitehead C. Pte. |

Wilson D. Sgt. Whitworth N. Copl.

*Arthur Ingham.]* *[Garden Street, Bury.*

is a beautiful example of the designer's skill and is framed in oak and gilt. The soldiers kept their intentions a secret until a few hours before the presentation took place, and it was a pleasant surprise to all concerned. We believe that this creates a precedent. Boards of management receive letters of gratitude, but illuminated addresses are rarely, if ever, given to them. This makes the address the more remarkable, and we are glad to reproduce an illustration of it.

### A BLOT ON THE 'SCUTCHEON.

THE sums contributed by the workmen of Newcastle and Leicester to their local hospitals are always interesting, since the workpeople's collections are looked to as models in less highly organised areas. Last year at Newcastle their collection reached £30,000, and, but for the grave fact that the Royal Infirmary there could, we learn, have filled its beds three times over, the year must be regarded as singularly successful. Partly because of several legacies, it ended with a balance in hand of £20,000. A sum of £11,000 was received from the military authorities. The Newcastle Orthopædic Centre is to be the chief consideration, and for this, as we note elsewhere, a donation of £10,000 is expected from the Government. We wish that something could be done to reduce the waiting list, whose present dimensions must be set against the good work which is being done in other directions. So long as this remains unreduced the work of the Royal Infirmary is incomplete, and new extensions of activity cannot receive whole-hearted applause.

### ESSENTIALS TO HAPPINESS IN THE HOME.

THE British, and the English people especially, are not famous for their culinary ability and results. Indeed, in the last few decades the number of Englishwomen, who have neither taste nor inclination for housekeeping and food supplies, has increased to a lamentable extent, to the no small disadvantage of English households. We hold the opinion that women of this type ought never to think of marriage, because they are not suited to family life in the highest and best meaning of the term. It is not a question, as some of the women we have in mind hold, of being household drudges at all. On the contrary, it is the proud privilege of the mother of a family to run her household with knowledge, and to organise it upon lines which secure good health and the comfort of its members. Without knowledge and intelligence in the mastery of the points which collectively ensure these results, the mother who is out of touch with the realities of everyday life and its necessities in the home has to leave such matters to servants, who speedily realise this to be so. Then they usually take advantage or become careless and inefficient in the discharge of their duties. How many homes have been broken up or rendered well-nigh impossible for some of their inmates where the mother is incompetent or neglectful or ignorant of the business of housekeeping and the smooth working of the home!

### RETIREMENT OF A HOSPITAL SECRETARY.

IT does not fall to the lot of every small town to have a resident taking such an interest in the local cottage hospital as to hold the office of honorary secretary for thirty years. At East Grinstead, however, this excellent record is now only being broken through the retirement to Eastbourne of Mr. H. A. Perkins, the honorary secretary of the hospital. A cheque amounting to £255 has been given to him in "warm appreciation of the energy, ability, and unfailing courtesy with which he has managed the affairs of the hospital." One who has had the opportunity of seeing his work in connection with the hospital can place on record not only the beautiful way in which the books were kept, but the accuracy which marked the minutest detail. Such painstaking work goes such a long way towards ensuring the smooth working of our small hospitals. Mr. Perkins will be missed at East Grinstead.

### THE FEEDING AND CARE OF INFANTS.

At the instance of the Plunket Society, of which the Right Hon. Lord Plunket is the chairman, Dr. Truby King, C.M.G., has come to London, with the sanction of the New Zealand Government, to lecture and demonstrate on the Feeding and Care of Infants. Pending the completion of arrangements which are being made by the Society for Dr. King to illustrate his methods, he has arranged, with the permission of the Society, to give a series of lectures and demonstrations in the Governors' Hall at St. Thomas's Hospital. These will be open to members of the Medical Profession, to Students, Nurses, and Infant Welfare workers. The first lecture will be given on Friday, May 3, at 5.30 p.m., when the Hon. Sir Arthur Stanley, M.P., the Treasurer of the hospital, will preside. The lectures will be repeated on Tuesdays and Fridays, at 5.30 p.m., for a course of five weeks. As these lectures are likely to be well attended, any who wish to go should at once make an application for a card of admission to the Secretary, Mr. G. Q. Roberts, at St. Thomas's Hospital.

### MR. GUY ELLISTON'S DEATH.

At the comparatively early age of forty-six Mr. Guy Elliston, the secretary of the British Medical Association, has died, after nineteen years' work for that body. The son of a medical man, he was educated at Ipswich, where he was born, and then went to Liverpool, where he engaged in the shipping industry. His way to his final post was begun when he became manager of the *Public Health Magazine*, after which, in 1898, he became assistant secretary to the B.M.A. and manager of the Association's journal. He was appointed secretary of the Association in 1902.

### THE MANCHESTER RADIUM INSTITUTE.

The committee of the Manchester and District Radium Institute has decided to purchase an additional supply of radium at a cost of some £2,400. A further sum of £300 is to be spent on the apparatus for research required by the laboratory, which is nearly completed. Sir William Milligan is to report on the subject, but it is understood that radium can be obtained at Pittsburg at a reasonable price.

### PRIZES FOR POSTERS BY THE WOUNDED.

Last Sunday, at the Kitchener House Club, which, under the British Red Cross Society and the Order of St. John, provides interesting occupations for the wounded, prizes were awarded in the poster competition arranged by the club for men who have been wounded on active service since August 1914. Lady Barker's prize of £10 for the best poster illustrating the work of the club was won by Mr. S. Knight, of 68 Torrington Square, a discharged man, who was formerly a lance-corporal in the Royal Engineers. The second prize, of £5, given by Mrs. Inglefield, was won by Private F. Dickinson, London Regiment, Clacton. The winner of the third prize, also of £5, given by Mrs. John Galsworthy, was Private C. L. Stephen, Welsh Regiment. This last prize, which was offered to men who have had no training in art, was for the best idea, irrespective of execution. Mr. Bernard Partridge was one of the judges. The clubhouse is at 8 Cambridge Gate, Regent's Park. There are no fees of any kind. All wounded men are welcome, and daily classes are held in various subjects, except on Saturday afternoons, when concerts are arranged.

### THE PAPER CONTROLLER'S ANXIETY.

We are asked to state that the Paper Controller is anxious for public assistance in preventing the waste of paper. Readers who come across instances of waste of paper should communicate by letter with the Paper Controller, 23 Buckingham Gate, S.W. Readers who may be in doubt as to what is wasted paper may gain advice by consulting their waste-paper baskets, which will put them on the track of many forms of extravagance.

### THIS WEEK'S DRUG MARKET.

The main event of the week has, of course, been the increases in taxation, which will affect the price of scores of medicinal preparations unless a rebate is allowed. With the duty on spirit doubled it is not difficult to estimate the increase in the cost of making tinctures and other spirituous preparations, and it is a fortunate thing that the concession with regard to spirit used in hospitals is in force; otherwise the increased taxation would have added substantially to the burden. The duty on saccharin has been increased proportionately to the duty on sugar; so far as British-made saccharin is concerned this increase does not affect saccharin issued to the trade before the date of the Budget. This will be sold at the prices printed on the labels on the tablet bottles, and the prices of tablets issued subsequent to the Budget will be announced in due course. Dealers in imported saccharin have already added the extra tax to their selling-prices, even though the extra duty has not been paid. In the drug market generally the upward tendency of prices continues, and in the case of a number of articles the scarcity is becoming more acute. Olive oil, liquid paraffin, and hard and soft paraffins are in very short supply. Cascara sagrada, ergot of rye, and senega root are dearer. The demand for bromides is quiet at the moment, and there has been no further advance in prices. As we go to press we understand that a rebate will be allowed on the duty on spirits used for medicinal preparations.

### TO OUR READERS.

**Contributions are specially invited from any of our readers to these columns. They should deal with topical subjects and news. They must be authenticated for the information of the Editor only. The minimum payment if published is 5s. There is no hard-and-fast rule as to space, but notes of about twenty lines in length are preferred.**

## Figures and Tables

**Figure f1:**